# Hemophagocytic lymphohistiocytosis and macrophage activation syndrome: two rare sides of the same devastating coin

**DOI:** 10.1186/s42358-024-00370-2

**Published:** 2024-04-16

**Authors:** Flavio Sztajnbok, Adriana Rodrigues Fonseca, Leonardo Rodrigues Campos, Kátia Lino, Marta Cristine Félix Rodrigues, Rodrigo Moulin Silva, Rozana Gasparello de Almeida, Sandro Félix Perazzio, Margarida de Fátima Fernandes Carvalho

**Affiliations:** 1 Department of Pediatrics, Universidade Federal do Rio de Janeiro, Rio de Janeiro, Brazil; 2 Pediatric Rheumatology Division, Hospital Universitário Pedro Ernesto, Universidade do Estado do Rio de Janeiro, Rio de Janeiro, Brazil; 3 Rare Diseases Committee, Brazilian Society of Rheumatology (SBR), Rio de Janeiro, Brazil; 4 Instituto de Puericultura e Pediatria Martagão Gesteira, Universidade Federal do Rio de Janeiro, Rio de Janeiro, Brazil; 5 Hospital Universitário Antônio Pedro, Universidade Federal Fluminense, Niterói, Brazil; 6 Pediatric Rheumatology Committee, Sociedade de Reumatologia do Rio de Janeiro 2022–2024, Rio de Janeiro, Brazil; 7 Pediatric Rheumatology Division, Instituto de Puericultura e Pediatria Martagão Gesteira, Universidade Federal do Rio de Janeiro, Rio de Janeiro, Brazil; 8 Hospital Universitário Pedro Ernesto, Universidade do Estado do Rio de Janeiro, Rio de Janeiro, Brazil; 9 Division of Rheumatology, Universidade Federal de São Paulo (UNIFESP), Sao Paulo, Brazil; 10 Division of Pediatric Rheumatology, Universidade Estadual de Londrina (UEL), Paraná, Brazil

**Keywords:** Macrophage activation syndrome, Hemophagocytic lymphohistiocytosis, Cytokines, Juvenile idiopathic arthritis

## Abstract

Hemophagocytic lymphohistiocytosis (HLH) is a rare genetic hyperinflammatory syndrome that occurs early in life. Macrophage activation syndrome (MAS) usually refers to a secondary form of HLH associated with autoimmunity, although there are other causes of secondary HLH, such as infections and malignancy. In this article, we reviewed the concepts, epidemiology, clinical and laboratory features, diagnosis, differential diagnosis, prognosis, and treatment of HLH and MAS. We also reviewed the presence of MAS in the most common autoimmune diseases that affect children. Both are severe diseases that require prompt diagnosis and treatment to avoid morbidity and mortality.

## Introduction

Cytokine storm syndrome (CSS) is a broad term encompassing various clinical conditions that involve hyperinflammation. Within this spectrum, hemophagocytic lymphohistiocytosis (HLH) is a rare yet highly fatal clinical condition if not diagnosed and treated promptly. For educational purposes, HLH can be classified as primary (familial) or secondary, related to various triggers such as infections, autoimmune diseases, malignancies, and inborn errors of immunity (IEI) [1].

Primary or familial HLH (FHL) commonly manifests in early childhood and results from mutations affecting key genes, necessary to the exocytosis pathway that rely on secretory lysosomes. Natural killer (NK) cell cytotoxic activity impairment was observed in patients with primary HLH in the 1980s. The first gene identified in primary HLH, PRF1, was described in December 1999 and is associated with familial HLH type 2. Other genes currently associated with primary HLH are *UNC13D, STXBP2, STX11, RAB27A, LYST, FAAP24, SCL7A7, RHOG, CEBPE, AP3D1* and *AP3B1*. Secondary HLH is more common in older children and adults [1–3].

While the term macrophage activation syndrome (MAS) is frequently used in patients with secondary HLH associated with rheumatic diseses, MAS and primary HLH share clinical and laboratory characteristics, genetic factors, and underlying mechanisms of pathogenesis. MAS is a clinical condition characterized by cytopenia, organ dysfunction, coagulopathy, and excessive macrophage activation and occurs in patients with hyperinflammation. The most common rheumatic diseases associated with MAS are systemic juvenile idiopathic arthritis (JIA), systemic lupus erythematosus (SLE), adult-onset Still disease (AOSD), Kawasaki disease, and autoinflammatory syndromes [4, 5].

**Fig. 1:**
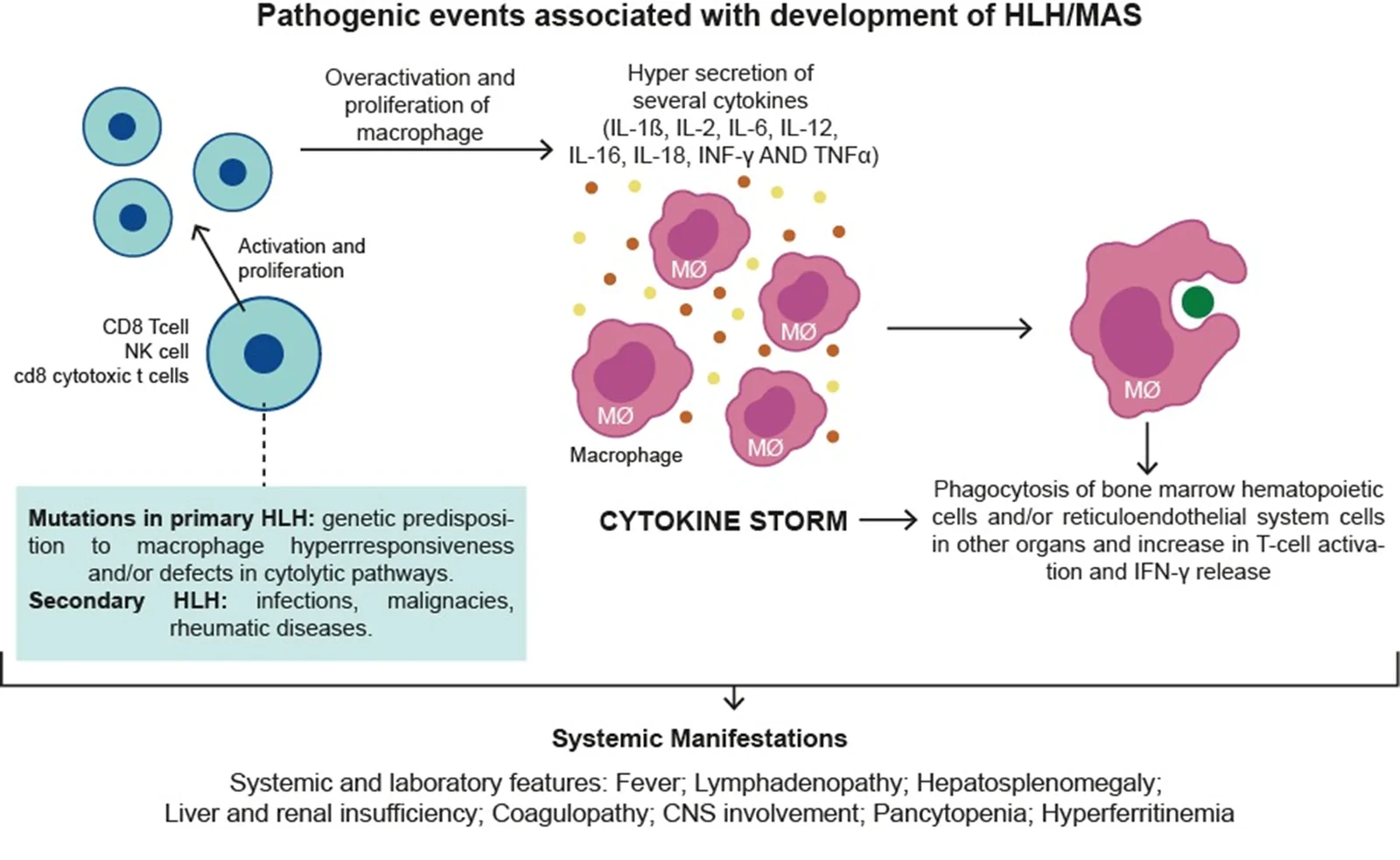
Pathogenic events associated with development of HLH/MAS

The initial concept that mutations in genes are exclusively found in primary HLH has evolved. Numerous patients with secondary HLH have been diagnosed with mutations in at least one allele of genes associated with familial HLH. Consequently, it is now understood that HLH is multifactorial and involves genetic and environmental contributions with varying degrees of influence, leading to different clinical presentations [6]. On the other hand, primary HLH pathogenesis involves a genetic defect in the perforin/granzyme pathway or the fusion of cytotoxic lytic granules with NK cell surfaces. Therefore, individuals with IEI impacting granule movement or exocytosis, such as Hermansky-Pudlak syndrome type 2, Griscelli syndrome type 2, and Chediak-Higashi syndrome, are at increased risk of developing HLH due to diminished cytotoxic T lymphocyte (CTL) functionality. Recent advancements in genetic diagnosis indicate a spectrum of CTL and NK cell capabilities for destroying cells, ranging from mild to severe, which helps explain the varied phenotypes of HLH. Mutations in genes responsible for granule-based killing are linked to both FHL and other primary forms [7]. Under physiological conditions, the interaction of NK cells and CTLs with a target cell triggers the development of secretory lysosomes. The lysosomes released carry toxic proteins such as perforin and granzymes [8]. When stimulated by external factors, such as viral infections, patients with genetic defects trigger an excessive reaction from cytotoxic CD8+ T lymphocytes, which in turn release substantial quantities of IFN-γ, leading to macrophage activation. In response, overactive macrophages produce inflammatory cytokines, including IL-1β, IL-6, IL-12, IL-18, and TNF-α, as well as increase IL-10 production, which has inhibitory effects but is insufficient for controlling this process. Additionally, IL-12 and IL-18 from macrophages further stimulate CD8+ T cells, exacerbating the inflammatory reaction. This cycle of inflammation results in tissue damage and the subsequent release of IL-33 and IL-1β, which further stimulate macrophages. Hyperactivated macrophages engulf blood cells (hemophagocytosis) and produce high levels of ferritin (a laboratory marker of the cytokine storm). The resulting ‘cytokine storm’ is responsible for various clinical manifestations of HLH, ranging from endothelial damage to coagulopathy and multiorgan failure [9]. Figure 1 presents a simplified diagram of the complex pathophysiology of HLH/SAM.

Recent advancements have seen a growing implementation of biomarkers, such as serum IL-18 and CXCL9, in medical practice. Specifically, CXCL9 has become instrumental in assessing IFN-γ activity during HLH and MAS, particularly in trials involving IFN-γ-inhibiting agents such as emapalumab. IL-18 has been independently demonstrated to be an effective reliable indicator of disease progression in MAS patients linked to active systemic JIA [1].

Regardless of the etiology, the resulting cytokine storm induces systemic inflammation with multiorgan failure. Hence, HLH should be considered a clinical syndrome of hyperinflammation with different phenotypes [10].

## Epidemiology

HLH/MAS is a rare but likely underrecognized hyperinflammatory syndrome that can occur in any age group and is associated with high mortality rates among children (8–22%) and adults (~40%) [9]. In children, infection is the most common etiology, with a specific pathogen identified in more than 50% of new HLH/MAS cases. Up to 25% of reported cases of HLH/MAS are genetic, 70–80% of which typically occur within the first year of life [9]. Acute infections are identified as the trigger of CSS in patients with primary HLH, as in those carrying IEI (>80%) or MAS or those with predisposing rheumatic conditions (>65%) [11]. The list of infectious pathogens is extensive and diverse (viral, bacterial, parasitic, and fungal) and influenced by geographic region (leishmaniasis and tick-borne illnesses), season (influenza viruses, tick-borne illnesses), and socioeconomic status (tuberculosis). The most common infectious agents include DNA viruses (Epstein-Barr virus (EBV), cytomegalovirus, and adenovirus) and intracellular pathogens (e.g., *Leishmania* sp.) [11]. Visceral leishmaniasis (VL) adds an extra diagnostic challenge in relation to MAS, considering that both conditions can present similar clinical and laboratory findings. On the other hand, VL can often trigger SAM secondary to infection by the parasite itself. and can manifest with intermittent high fever, anorexia, weight loss, hepatosplenomegaly, lymphnode enlargement, pancytopenia, hypoalbuminemia, and hypergammaglobulinemia. It should be noted that the parasite can carry out part of its cycle by infecting macrophages and, multiplying there, evading the microbicidal power of the macrophage phagolysosome, finding a place to develop and multiply. Studies suggest that the parasite actively induces hemophagocytosis, using parasitized red blood cells as a source of iron [12, 13].

In the context of rheumatic diseases, systemic JIA is the most common cause and MAS occurs clinically in approximately 10% of patients, with subclinical or partial presentations in 30–40% of children [14, 15].

Malignancy-associated hemophagocytic lymphohistiocytosis is a hyperinflammatory syndrome that carries a very bad prognosis and, together with infection-associated HLH, are the most common forms of secondary HLH. In adults, malignancy may be the contributing factor to HLH/MAS in 50% of patients. Lymphoma and leukemia are common malignancies associated with HLH/MAS, particularly T cell, NK cell, diffuse large B cell, and Hodgkin lymphoma [15]. A recente Swedish study showed that, among the malignancies, 52% were lymphomas, 29% leukemias, 8% other hematological malignancies, and 11% solid tumors. Two ways of presentation can occur: either malignancy-triggered HLH, in which HLH typically is present before or concomitantly with the diagnosis of the malignancy, or HLH occuring during chemotherapy, usually triggered by infections [16]. An example is the Chimeric antigen receptor T cell–associated HLH (CAR HLH), a kind of citokyne release syndrome (CRS) toxicity secondary to immune system activation and inflammation, reported in patients receiving CD-19 and CD-22 specific CAR T cell therapy for leukemia/lymphoblastic lymphoma. CAR HLH occur most commonly in patients with high disease burden and clinical and laboratory picture resemble classic HLH/MAS manifestations [17, 18]. It has been observed an increase in short-term survival likely due to increased awareness of HLH and earlier treatment [19].

## Clinical manifestations

HLH can affect all organ systems, although a single manifestation is not specific, and a wide range of diseases may display similar findings. In addition, early-stage HLH/MAS can be highly variable among patients and often involves rapid changes within the same patient. The presence of unexplained persistent high fever, hepatosplenomegaly, cytopenias (absolute or relative compared to baseline levels), liver dysfunction, and elevation of typical HLH/MAS biomarkers, especially if unresponsive to initial antibiotics, should lead to high diagnostic suspicion [15]. Furthermore, patients may have neuropsychiatric findings, lymphadenopathy, fatigue, anorexia, headache, rash, diarrhea, arthralgia, and myalgia. Neurological manifestations at disease onset have been reported in up to 30% of patients and include irritability, depressed consciousness, hypotonia, cranial nerve palsies, ataxia, and seizures. In neonates, HLH/MAS may present with isolated central nervous system (CNS) involvement or with fulminant liver failure. Patients are often very unwell and can quickly progress to hemodynamic instability, disseminated intravascular coagulation, acute respiratory distress syndrome, renal failure, acute liver injury, multiorgan failure, and death. The combination of renal dysfunction and acute-phase hypoalbuminemia can lead to capillary leak syndrome and anasarca. The clinical features may be challenging to distinguish from flares of the underlying disease or from sepsis. Therefore, a multidisciplinary approach is required to optimize the diagnostic workup and management of these patients [11, 15].

## Laboratory findings

The European Alliance of Associations for Rheumatology (EULAR)/American College of Rheumatology recently created a multinational, multidisciplinary task force of experts to develop overarching statements and specific points to help guide the initial evaluation, management and monitoring of patients with HLH/MAS based on the best available published data and expert opinions [11]. Once a diagnosis of HLH/MAS is suspected, laboratory and imaging studies should be performed to gather supportive evidence for the diagnosis and to assess organ involvement and severity. In addition to a compatible clinical phenotype, the most important point to keep in mind is that sequential laboratory biomarker assessment is more critical than absolute timepoint values for an earlier suspicion and diagnosis of HLH/MAS [20]. Patients may present not only absolute but also relative cytopenia (leukopenia, neutropenia, anemia, thrombocytopenia), and evolution in comparison to baseline counts is required. Notably, HLH/MAS can occur even with unexpectedly normal counts but in the face of active systemic inflammation, similar to systemic JIA (sJIA). C-reactive protein (CRP) is universally elevated and correlated with disease severity. The paradoxically decreasing erythrocyte sedimentation rate (ESR) in the setting of systemic inflammation is thought to be secondary to decreasing fibrinogen due to consumptive coagulopathy and liver dysfunction [20]. Ferritin is a sensitive test for HLH/MAS, and there is consensus that ferritin levels should be checked in all patients with new, ongoing, or heightened suspicion even if prior measurements have been normal. Ferritin levels also have prognostic relevance, as both higher initial ferritin levels and failure to improve during therapy are associated with worse outcomes [11]. A significant increase in the serum ferritin concentration (e.g., greater than 10,000 ng/ml) in the setting of a hospitalized febrile patient is a simple screening tool for HLH/MAS. Other laboratory abnormalities include hypertriglyceridemia, elevated d-dimer levels, high or increasing transaminase and lactate dehydrogenase (LDH) levels, and low or decreasing fibrinogen [20]. Pleocytosis accompanied by an increased protein level in the cerebrospinal fluid or abnormal radiological findings detected by computed tomography (CT) or magnetic resonance imaging (MRI) can sometimes be observed at disease onset or during the course of the disease, despite the absence of neurological abnormalities [21]. The use of cytokine-targeted biologics against interleukin-1 and interleukin-6 poses another challenge, as it has been suggested by some studies that they might mask MAS symptoms and laboratory data [22].

More specialized inflammatory biomarkers of HLH/MAS pathways, such as IFN-γ (or CXCL9 and CXCL10), IL-6, TNF-α, IL-18, soluble interleukin-2 receptor alpha, NK cell activity, and CD163/neopterin (marker of macrophage activation), are usually abnormal [23].

The genetic causes of HLH/MAS are likely underrecognized, and a positive finding has a large impact on treatment, prognosis and genetic counseling. Therefore, genetic testing in children and high-risk adults with suspected or confirmed HLH/MAS should be considered early, preferably using multigene panels or whole-exome/genome sequencing.

## Diagnosis

There is no single pathognomonic feature or diagnostic marker for HLH/MAS, and early diagnosis requires a high index of suspicion relying on a combination of clinical features and laboratory findings.

The Histiocyte Society and later rheumatology consortia developed and refined classification criteria to define HLH or MAS. Subsequent diagnostic tools, such as the HScore and MAS/sJIA score, provide quantitative information [11]. However, those existing criteria perform well in the specific settings from which they were derived, and no single set of criteria is sufficient to diagnose HLH/MAS across all contexts. The HLH-94 criteria (refined in HLH-04) were developed to classify infants and children for trials targeting pediatric patients with genetic causes of HLH, requiring 5 of 8 criteria to be met (Table 1) [24]. A major problem with the HLH-2004 criteria is that measurements of NK cell function and soluble interleukin 2 receptor α chain (sIL-2Ra, CD25) are available only in very few laboratories.

**Table 1:** Revised diagnostic guidelines for hemophagocytic lymphohistiocytosis [24]

**The diagnosis HLH can be established if one of either 1 or 2 below is fulfilled:**		
(1) A molecular diagnosis consistent with HLH (2) Diagnostic criteria for HLH fulfilled (5/8 criteria below)		
**(A) Initial diagnostic criteria** (to be evaluated in all patients with HLH)		
	**1. Fever**	
	**2. Splenomegaly**	
	**3. Cytopenias** *(affecting 2 of 3 lineages in the peripheral blood):*	
		Hemoglobin < 9 g/dL (in infants < 4 weeks: Hb < 10 g/dL); Platelets < 100,000/mm³; Neutrophils < 1000/mm³
	**4. Hypertriglyceridemia and/or hypofibrinogenemia:**	
		Fasting triglycerides 3.0 mmol/L (i.e., 265 mg/dl); Fibrinogen ≤ 1.5 g/L
	**5. Hemophagocytosis in bone marrow or spleen or lymph nodes**	
		**No evidence of malignancy**
**(B) New diagnostic criteria**		
	**6. Low or absent NK-cell activity** (according to local laboratory reference)	
	**7. Ferritin** ≥** 500 mg/L**	
	**8. Soluble CD25** (i.e., soluble IL-2 receptor) ≥ **2400 U/ml**	
Comments: (1) In cases where hemophagocytic activity is not initially evident, it is recommended to conduct further investigations to confirm its presence. If the examination of bone marrow samples yields inconclusive results, samples from alternative organs may be sought. Additionally, serial marrow aspirates over a period of time could offer valuable insights (2) The following observations may strongly support the diagnosis: (a) presence of pleocytosis (mononuclear cells) and/or elevated protein levels in cerebrospinal fluid, (b) hepatic histology resembling that of chronic persistent hepatitis as observed in liver biopsies (3) Additional clinical and laboratory abnormalities consistent with the diagnosis include: symptoms related to the central nervous system and meninges, enlargement of lymph nodes, jaundice, edema, and skin rash. Abnormalities in hepatic enzymes, hypoproteinemia, hyponatremia, elevated VLDL, and decreased HDL may also be present		

In 2016, an expert consensus panel published a set of validated classification criteria to help distinguish sJIA flares from MAS. MAS can be identified in a febrile patient with or suspected of having sJIA with relatively few total criteria with laboratory data routinely available everywhere (Table 2) [5, 25].

**Table 2:** Classification [5] and diagnostic [25] criteria for macrophage activation syndrome (MAS) in systemic juvenile idiopathic arthritis

Parameter	**2016 sJIA/MAS** [5]	MAS/sJIA score [25]
Fever	Not specified	–
Ferritin	>684 ng/mL	0.0001* serum level
Platelet count	Platelets ≤ 181,000/mm³	−0.003* platelet count
Hemorrhagic manifestations	–	1.54*1 (yes) or *0 (no)
Fibrinogen level	≤360 mg/dL	−0.004* serum level
LDH	–	0.001* serum level
AST	>48 units/L	–
Triglycerides	>156	–
Central nervous system	–	2.44 *1 (yes) or *0 (no)
Active arthritis	–	−1.3 *1 (yes) or *0 (no)
Diagnosis:	Ferritin >684 + 2 criteria (platelet, AST, triglycerides, or fibrinogen)	Sum of parameters ≥ −2.1

*LDL* Lactate dehydrogenase level, *AST* Aspartate aminotransferase

CSS encompasses many conditions that may even be concomitant with or mimic HLH/MAS. Infection is a common cause of CSS, and it may appear as an isolated trigger or be associated with other inflammatory conditions. Bacterial sepsis and others infectious agents are generally involved, such as intracellular pathogens, herpesviruses, hemorrhagic fever virus (e.g., dengue fever), severe acute respiratory syndrome coronavirus 2 (SARS-CoV-2) and HIV/AIDS. Moreover, ruling out malignancy-associated HLH, malignant histiocytic disorders, tumor lysis syndrome and drug reactions is highly recommended [14, 26–29]. In the context of flares resulting from an underlying inflammatory or autoimmune condition, especially sJIA, adult-onset Still disease or SLE, clinical and laboratory issues such as hepatic dysfunction, coagulopathy, encephalopathy, cytopenia and hyperferritinemia should provide diagnostic clues. Hyperferritinemia may also be associated with liver, kidney and hematological disorders [14, 15, 20–24, 26]. Although rare, genetic causes of HLH/MAS must be considered in seriously ill pediatric patients [1–4].

Considering that clinical features are similar to those of someinfections and inflammatory disorders and that molecular geneticdiagnosis for familial HLH is not easily available everywhere and and may take a long time to accomplish, flow cytometric assays may be used as a firstdiagnostic approach in some forms of familial HLH, as a faster andmore cost-effective tool for initial diagnosis and functionalvalidation. In recent years, assays based on flow cytometry have beendeveloped for evaluating NK cell and cytotoxic T lymphocytes functionsthat may reflect functional deficits in key proteins that play a majorrole in lymphocyte cytotoxicity. Granule release assay (GRA) is ascreening test for detection of FHL3, FHL4, and FHL5 patients.Measurement of intracellular perforin levels serves as a phenotypic assay in identifying FHL2 patients [21, 30].

## Histopathology

Despite the name of this condition, the observation of hemophagocytosis is not required for a diagnosis of HLH/MAS. While the presence of characteristic increased hemophagocytic activity with positive CD163 (histiocyte) staining and hemophagocytosis in the bone marrow can help confirm the diagnosis, only 30% to 60% of patients will have this finding at early stages [26, 27]. Moreover, bone marrow biopsy may be inappropriate for critically unwell patients. The most expected finding is bone marrow hemophagocytosis, the engulfment of erythrocytes, lymphocytes or other hematopoietic precursors by histiocytes or macrophages that may also be present in the lymph nodes, liver or spleen. The histopathological finding is tissue infiltration with T lymphocytes and active macrophages with hemosiderin deposits and degenerating cells [28].

Nonetheless, the presence of hemophagocytosis has variable sensitivity and poor specificity, as it may be absent, especially in the early stages [24, 27, 28]. Although hemophagocytosis is not an absolute condition for HLH/MAS diagnosis, bone marrow examination is recommended to exclude malignancies and infection in all patients with suspected HLH/MAS.

## Treatment

HLH/MAS is a life-threatening condition that requires prompt recognition, immediate therapeutic intervention, systemic inflammation control (underlying disease and/or elimination of triggers), protection of organ function, and minor toxicity. Dynamic management is imperative and involves supportive care; an infectious workup including empiric and prophylactic therapies; immunomodulation; and immunosuppression. Simultaneously, several etiologies must be investigated with continuous monitoring and reassessment. The choice of treatment should be based on the available evidence and tailored to each patient considering the cause of CSS, contribution of host genetics, acute environmental triggers, severity, and heterogeneity of clinical manifestations. If a genetic cause of HLH/MAS is suspected, specific management may be necessary, and hematopoietic stem cell transplantation (HSCT) may be curative after controlling systemic inflammation [11, 31]. The etoposide-based treatment protocol HLH-94 consists of 8 weeks of induction therapy and subsequente continuation therapy until HSCT for patients with familial, relapsing, or refractory HLH. The subsequente HLH-2004 protocol confirmed this efficacy and showed that addition of cyclosporin (CSA) and intrathecal corticosteroids did not improve results. Morbidity and mortality of patients with HLH remained significant in these studies, achieving a 5-year survival of 54% and 65% respectively. Following studies mainly targeting pediatric patients with familial HLH and patients without underlying infectious, inflammatory or malignant disease, the HLH-94 protocol was recommended as the standard of care for HLH, but with careful guidance, in particular, if used beyond the indications of the HLH-94/2004 study protocols. Further in 2018, the HLH Steering Committee of the Histiocyte Society published recommendations regrading the use of the HLH-94 protocol, based on a structured consensus process and on expert opinion supported by literature available data. The severity and progression of disease manifestations rather than the fulfillment of the HLH criteria per se are critical for the decision to initiate the HLH-94 protocol. Also, HLH-94 therapy can be indicated in patients with primary HLH who present with isolated CNS disease. Patients with primary HLH carry a high and lifelong risk of reactivation, even after control of the acute HLH episode. Allogeneic HSCT is currently the only option for long-term cure in primary HLH. Therefore, early referral and shared decision making processes with an HSCT expert should begin soon after a diagnosis of primary HLH. In patients with primary HLH, 8 weeks of induction should be followed by continuation therapy as a brigde until HSCT, although there is no evidence whether it will prevent reactivation/relapse. Treatment of malignancy-associated hemophagocytic is similar to other forms of HLH, consisting of immunoglobulins, corticosteroids, and/or cyclosporine A, and some patients need to receive the more agressive HLH protocol treatment with dexamethasone and etoposide [19, 32]. Tables 3 and 4 summarize the main currently available immunosuppressant therapies for rheumatic diseases associated with MAS [17, 29, 31–42].

**Table 3:** Empirical immunomodulatory therapeutics for rheumatic diseases associated with MAS [11, 31–34]

Therapeutic agent	Target/action	Dosing
Glucocorticoids [11, 33]	Broad immunosuppression	a- Methylprednisolone IV or PO prednisone 1–2 mg/kg/day b- Methylprednisolone IV 10–30 mg/kg/day (maximum 1 g/day for 3 follow-up days) c- Dexamethasone IV/PO 10 mg/m²/day
Anakinra (rhIL-1Ra) [11, 33, 34]	Blocks IL-1 receptor binding	5–10 mg/kg/day IV/SC q6-12 hr
Intravenous immunoglobulin (IVIG) [11, 33]	Multiple targets	1–2 g/kg/day × 2 days IV

*IV* Intravenous, *mg* Miligrams, *PO* Oral use, *SC* Subcutaneous, *rhIL-1Ra* Recombinant human IL-1 receptor antagonista, *IVIG* Intravenous immunoglobulin

**Table 4:** Other therapies for HLH/MAS [11, 29, 31–33, 35–42]

Therapeutic agent	Dosing	Target	Action	Adverse events
Etoposide [11, 32, 35] (topoisomerase II inhibitor)	50–150 mg/m²/dose/week IV	T lymphocytes	Inhibits cell proliferation	Bone marrow suppression, hepatotoxicity, nephrotoxicity, mucositis, alopecia, secondary malignant hypotension
Ciclosporin [11, 33, 35] (calcineurin inhibition)	3–7 mg/kg/day q12 hr PO	IL-2, IFNγ, others	Inhibits cell proliferation and effector functions	Nephrotoxicity, hypertension, hepatotoxicity, neurotoxicity, hirsutism, gingival hypertrophy
Rituximab [11, 36, 37] (anti-CD20 mAb) *EBV-MAS	375 mg/m²/dose (maximum 1 g) q15 days IV or 375 mg/m²/dose (max 1 g) q7 days up to 4 consecutive weeks IV or 750 mg/m²/dose (max 1 g) q15 days IV	B lymphocytes	Depletes B lymphocytes	Infusion reactions, hepatotoxicity, nephrotoxicity, hypertension, immunosuppression, cytopenia, IgG, progressive multifocal leukoencephalopathy
Emapalumab [38] (anti-IFNγ mAb) *Refractory HLH	1–10 mg/kg/dose And then 3 mg/kg/dose Every 3 days IV	IFNg	Neutralizes IFNg	Immunosuppression (mycobacteria, herpesviruses and *Histoplasma capsulatum*), infusion reactions, hypertension
Ruxolitinib [39] (JAK 1/2 inhibition)	2.5–20 mg/dose or 25 mg/m²/dose q12 hr PO	IFNγ, IL-6, IL-12 and others	Inhibits cytokine signaling	Immunosuppression (herpesviruses), dyslipidemia, hepatotoxicity, cytopenia
Plasmapheresis [40]		Multiple cytokines	Removes proinflammatory mediators	Allergic reactions, fever, infections

Salvage therapies: Anti-thymocyte globulin (ATG) [41] and alemtuzumab (anti-CD52 mAb) [42]

*IV* Intravenous, *mg* Miligrams, *PO* Oral use, *SC* Subcutaneous, *IL* Interleucin, *IFN* Interferon, *mAb* Monoclonal antibody

### Supportive therapy

Intensive care support is required for 1/3 of children with HLH/MAS, and most of them need mechanical ventilation, vasopressors/inotropes, and renal replacement therapy. Strict fluid control, nutrition, blood product replacement for disseminated intravascular coagulopathy and drug adverse event and infectious intercurrence monitoring are strictly necessary [4].

### Acute triggers

Infection is the most common acute trigger of HLH/MAS and should be diagnosed and treated aggressively in all forms of HLH. Empiric broad-spectrum antimicrobial therapy is initially indicated [4]. In patients with EBV-driven HLH, B-cell depletion with rituximab improves the clinical parameters of the disease when used in combination with traditional HLH therapies. However, in some states of persistent EBV replication, EBV has been demonstrated to be expressed in T or NK cells, leading to resistance to rituximab treatment [14, 32, 35]. Etoposide-based therapy has been life-saving for patients with primary HLH and severe EBV-HLH but is not indicated for most non-EBV infections [43]. Other infections should be treated aggressively with antimicrobial agents, and in some cases, intravenous immunoglobulin (IVIG) may be used [29, 43]. In addition, during HLH/MAS treatment, prophylaxis against herpes zoster*, Pneumocystis jirovecii* and fungal infections should be considered according to individual comorbidities, chronic immunosuppression and pathogen exposure [11]. Immunosuppression and immunomodulation for the underlying condition are often required in patients with MAS induced by active autoimmune or autoinflammatory disease along with eradication of the infection [44].

### Empiric immunomodulation therapy

In all newly diagnosed patients, the search for underlying or associated conditions must be undertaken to choose the most effective treatment, as prompt control of systemic inflammation may prevent the development of severe CSS. Moreover, empiric immunomodulation of HLH/MAS should be initiated early to avoid severe immunosuppression that may compromise the etiological workup. Although no studies have evaluated empiric treatment for HLH/MAS prior to or regardless of etiology, immunomodulatory treatment has dramatically improved survival in most etiologies of HLH/MAS [11, 33].

The traditional treatment for rheumatic disease-associated MAS is glucocorticoids [11, 32]. If patients are resistant to corticosteroid therapy, cyclosporine could be added to the traditional treatment regimen [14]. IVIG neither obstructs cancer workup nor suppresses immune function and may be useful in the initial approach, especially in combination with other therapies.

In addition to broad immunosuppressants, immunomodulation with the cytokine-specific blocker anakinra, a recombinant IL-1 receptor antagonist that targets both the cytokines IL-1α and IL-1β, may prove to be very effective. Anakinra could be a promising therapy for nonmalignancy-associated HLH [45]. To date, the effects of other IL-1 inhibitors, including canakinumab (a monoclonal antibody that targets only the IL-1β cytokine) and rilonacept on MAS, have rarely been reported. Patients with sJIA treated with either anakinra or canakinumab are at dose-dependent risk for MAS, even those with fully controlled disease, suggesting that the IL-1 receptor is not the sole contributor to the pathogenesis of MAS, particularly in the setting of viral infection [22, 34]. Anakinra may also yield good results in treating sepsis-related and severe coronavirus disease (COVID-19-related CSS) [46].

IL-6 blockade with tocilizumab (an anti-IL-6R mAb) has proven effective in treating sJIA but is not protective against MAS development [22, 34]. Treatment with recombinant IL-18 binding protein (tadekinig alfa) may be a good option for patients with diseases that are primarily driven by inflammasome activation and high IL-18 levels [47]. The U.S. Food and Drug Administration (FDA) has also recently approved emapalumab (anti-IFN-γ) for primary/familial HLH in combination with dexamethasone and cyclosporine [4, 38, 48]. Ruxolitinib, a Janus kinase-1 and 2 inhibitor, has the advantage of targeting multiple cytokines, including IFN-γ. The inhibition of IFN-γ has been successfully used in refractory HLH/MAS and may hold promise for the treatment of HLH/MAS [39]. Therapeutic apheresis, including plasma exchange, leukocytapheresis, and plasma diafiltration, was recently reported to be effective at inducing disease remission, especially for patients with severe, refractory MAS, possibly by removing proinflammatory cytokines and activating inflammatory cells rapidly [40].

### Etoposide and refractory HLH/MAS

Historically, the first formal treatment for HLH consisted of dexamethasone and etoposide. Patients with CNS involvement received additional intrathecal treatment with methotrexate. Many patients are still treated with this approach [32, 35]. Etoposide is suggested to be administered in patients with refractory rheumatic disease-associated MAS. Various other therapeutic agents, such as cyclophosphamide, rituximab, anti-thymocyte globulin and alemtuzumab, which are anti-CD52 antibodies that deplete circulating B and T lymphocytes, have been used for refractory HLH/MAS [36, 41, 42].

## Prognosis

The prognosis of patients with HLH/SAM depends on multiple factors, including the underlying disease, organ dysfunction status, and disease activity duration. The condition can be fatal in any context, although patients with rheumatic diseases have a more favorable prognosis than patients with neoplasms [5, 11]. MAS can result in multiple-organ failure, with a mortality rate of approximately 8–22% [5, 11, 49]. Therefore, early recognition and immediate treatment are essential. The exact role of protective immunobiologicals for MAS is not yet known; however, some patients who use canakinumab or tocilizumab and develop this complication have been previously reported, suggesting that these drugs are ineffective prophylactically and that MAS may occur even in patients with controlled disease [50, 51]. The involvement of several targeted organs, such as the liver, can confer a worse prognosis and can rapidly progress to failure/insufficiency. Patients who require admission to the intensive care unit with multiple organ dysfunction also have a worse prognosis. CNS involvement is more common in children than in adults and can be insidious, dangerous, and associated with greater morbidity, mortality and neurological sequelae [11]. The following factors lead to a worse prognosis in MAS patients: late diagnosis, multiple organ dysfunction, severe neutropenia, coagulopathy, CNS disease and lack of response to treatment [11].

## MAS and the most prevalent pediatric autoimmune and autoinflammatory diseases

Although the prevalence of MAS among patients with JIA is estimated to be approximately 10%, some studies have reported that this prevalence can reach up to 30–40% [5]. Several criteria have been proposed for diagnosing MAS in patients with JIA, as reported previously, but there is still difficulty in differentiating cases of active systemic disease from those that evolve with MAS. It is speculated that JIA and MAS are two ends of the same spectrum, with the majority of patients being in the middle [5]. Patients using tocilizumab require special attention, as they present milder symptoms of MAS and do not experience progression with elevated CRP levels [50]. The serum IL-18 concentration increases before that of other markers and is very high during the recurrence of sJIA; moreover, the serum IL-18 concentration gradually decreases with immunosuppressive treatment, making it an excellent marker for monitoring risk in patients with sJIA [50]. In the review carried out by Minoia et al., 362 patients with JIA developed MAS; 34.9% required admission to the intensive care unit, while 8.1% died [52].

The estimated prevalence of MAS among patients with SLE is 0.9 to 4.6%. However, these values may be underestimated [53, 54]. Several studies suggest that MAS may be associated with more severe organic involvement and higher mortality among children [55]. In patients with SLE, the laboratory findings most strongly associated with MAS are hyperferritinemia, elevated LDH levels, hypertriglyceridemia and hypofibrinogenemia. Thrombocytopenia and leukopenia are more common in MAS, but cytopenia is common in both SLE and MAS patients with any etiology. Patients with SLE with persistent and unexplained fever associated with cytopenia and hyperferritinemia should be evaluated for the possibility of MAS [53]. Table 5 presents a proposed diagnostic criteria for MAS as a complication of juvenile systemic lupus erythematosus [14, 56]. There are few reports of MAS related to juvenile dermatomyositis, in which is considered an infrequent complication.

**Table 5:** Diagnostic criteria for macrophage activation syndrome (MAS) as a complication of juvenile systemic lupus erythematosus (SLE) [14, 56]

The diagnosis of MAS requires the simultaneous presence of 1 clinical criterion + at least 2 laboratory criteria. Bone marrow aspiration for evidence of macrophage hemophagocytosis may be required in doubtful cases. This diagnostic criteria may not be powerful enough to distinguish MAS from particular infectious complications
**Clinical criteria**
1. Fever (>38 °C)
2. Hepatomegaly (≥3 cm below the costal arch)
3. Splenomegaly (≥3 cm below the costal arch)
4. Hemorrhagic manifestations (purpura, easy bruising, or mucosal bleeding)
5. Central nervous system dysfunction (irritability, disorientation, lethargy, headache, seizures, or coma)
**Laboratory criteria**
1. Cytopenia affecting 2 or more cell lineages (white blood cell count ≤ 4000, hemoglobin ≤ 9 mg/dL, or platelet count ≤ 150,000/mm³
2. Increased aspartate aminotransferase (>40 units/L)
3. Increased lactate dehydrogenase (>567 units/L)
4. Hypofibrinogenemia (fibrinogen ≤ 1.5 g/L)
5. Hypertriglyceridemia (triglycerides > 178 mg/dL)
6. Hyperferritinemia (ferritina > 500 mcg/L)
**Histopathologic criterion**
Evidence of macrophage hemophagocytosis in the bone marrow aspirate

MAS can be diagnosed before, during or after the diagnosis of Kawasaki disease (KD), with most cases occurring later. The estimated incidence of SAM among KD patients is between 1.1 and 1.9%, with a 7-fold greater risk among patients older than 5 years. It is also more prevalent among males [54, 57, 58]. The clinical and laboratory findings of the two conditions overlap. The persistence of fever, associated with splenomegaly, thrombocytopenia, hyperferritinemia, elevated AST levels and lack of response to IVIG treatment, should raise the suspicion of MAS. In particular, the decrease in platelet count in patients with KD suggests a potential risk of progression to MAS [57, 58]. Different therapeutic approaches include immunoglobulin, corticosteroids, cyclosporine, etoposide, infliximab and anakinra [57–59]. Considering that the majority of patients with KD receive IVIG and corticosteroids with good control of the disease, it is postulated that the occurrence of MAS in these patients is underdiagnosed [58], in addition to the fact that some tests for the diagnosis of MAS are not routinely requested for patients with KD [58, 59]. Patients who present with recurrent or refractory KD should be investigated for the possibility of progression to MAS [57–59].

There are several reports of MAS related to autoinflammatory diseases, such as familial Mediterranean fever [60], A20 haploinsufficiency [61], periodic syndrome associated with the tumor necrosis factor receptor (TRAPS) [62], and deficiency of mevalonate kinase [63].

In summary, HLH and MAS may be considered two sides of the same coin; HLH can be considered the familiar or primary form of presentation and those forms related to infections and malignancies while MAS is the secondary form associated with autoimmune diseases. Both are severe in clinical presentation and laboratory features and require prompt diagnosis and treatment to avoid increased morbidity and mortality associated with “both sides of the coin”.

## Data Availability

Data sharing was not applicable. No new data were generated.

## Abbreviations

CSS: Cytokine storm syndrome
HLH: Hemophagocytic lymphohistiocytosis
IEI: Inborn errors of immunity
FHL: Familial HLH
NK: Natural killer
MAS: Macrophage activation syndrome
JIA: Juvenile idiopathic arthritis
SLE: Systemic lupus erythematosus
AOSD: Adult-onset Still disease
CTL: Cytotoxic T lymphocyte
CAR: Chimeric antigen receptor
CRS: Cytokine release syndrome
EBV: Epstein-Barr virus
CNS: Central nervous system
sJIA: Systemic juvenile idiopathic arthritis
CRP: C-reactive protein
ESR: Erythrocyte sedimentation rate
LDH: Lactate dehydrogenase
CT: Computed tomography
MRI: Magnetic resonance Imaging
AST: Aspartate aminotransferase
SARS-CoV-2: Severe acute respiratory syndrome coronavirus 2
HSCT: Hematopoietic stem cell transplantation
IVIG: Intravenous immunoglobulin
FDA: Food and Drug Administration
KD: Kawasaki disease
